# Human Cortical Neural Progenitor Cells

**DOI:** 10.1111/cpr.70266

**Published:** 2026-08-04

**Authors:** An‐Xin Wang, Jie Hao, Guang‐Zhu Zhang, Ai‐Jin Ma, Fang Ren, Yan‐Shuang Xiao, Yu Zhang, Guang‐Jin Pan, Tao Na, Ke‐Hua Zhang, Jun Yao, Ying Zhang, Yang Zhao, Yi Zhou, Shi‐Jun Hu, Hong‐Ling Zhao, Mei Tian, Hong Zhang, Li‐Jun Zhu, Qi‐Yuan Li, Jia‐Ni Cao, Ting‐Ting Wu, Qiang Wei, Wei‐Qi Zhang, Ka Li, Lei Wang, Shuai‐Shuai Niu, Zhi‐Quan Yang, Tong‐Biao Zhao, Jing Fan

**Affiliations:** ^1^ Hopstem Bioengineering Co., Ltd. Hangzhou China; ^2^ National Stem Cell Resource Center, Institute of Zoology, Chinese Academy of Sciences Beijing China; ^3^ Standard Committee, Chinese Society for Cell Biology Shanghai China; ^4^ Chinese PLA General Hospital Beijing China; ^5^ Beijing Technology and Business University Beijing China; ^6^ Zephyrm Biotechnologies Co., Ltd Beijing China; ^7^ Guangzhou Institutes of Biomedicine and Health, Chinese Academy of Sciences Guangzhou China; ^8^ National Institutes for Food and Drug Control Beijing China; ^9^ Tsinghua University Beijing China; ^10^ Nuwacell Biotechnologies Co., Ltd. Hefei China; ^11^ Peking University Beijing China; ^12^ State Key Laboratory of Neuroscience Institute of Neuroscience, CAS Center for Excellence in Brain Science and Intelligence Technology, Chinese Academy of Sciences Shanghai China; ^13^ Soochow University Suzhou China; ^14^ Beijing Institute for Stem Cell and Regenerative Medicine Beijing China; ^15^ Fudan University Shanghai China; ^16^ Zhejiang University Hangzhou China; ^17^ Institute of Scientific and Technical Information of China Beijing China; ^18^ Shenzhen Medical Academy of Research and Translation Shenzhen China; ^19^ Institute of Laboratory Animal Science Chinese Academy of Medical Sciences Beijing China; ^20^ China National Center for Bioinformation and Beijing Institute of Genomics, Chinese Academy of Sciences Beijing China; ^21^ Zhongshan Hospital, Fudan University Shanghai China; ^22^ Institute of Zoology, Chinese Academy of Sciences Beijing China; ^23^ Xiangya Hospital of Central South University Changsha China

The standard, human cortical neural progenitor cells, is developed through discussions and mutual agreements among experts from academia, industry, clinical practice, regulatory bodies, independent third parties and members of the Chinese Society for Stem Cell Research. It is jointly drafted and agreed upon by experts from the Standard Committee of the Chinese Society for Cell Biology. This guideline specifies general and technical requirements, inspection methods and rules, instructions for use, labelling, and storage and transportation requirements for hCNPCs. It was originally released by the China Society for Cell Biology on 28th October 2024. The aim of publishing this guideline is to facilitate non‐clinical and clinical developments of hCNPCs with appropriate protocols and to accelerate their therapeutic applications of hCNPCs.

## 1 Scope

This document specifies the general requirements, technical requirements, inspection methods, inspection rules, instructions for use, labelling, storage and transportation requirements for human cortical neural progenitor cells.

This document is applicable for the inspection of human cortical neural progenitor cells derived from human tissue separation, stem cell differentiation or trans‐differentiation.

## 2 Normative References

The following content constitute indispensable articles of this standard through normative reference. For dated references, only the edition cited applies. For undated references, only the latest edition (including all amendments) applies.

WS 213/T *Diagnosis for Hepatitis C Virus*.

WS 293/T *Diagnosis for HIV/AIDS*.

WS 298 *Diagnosis for Hepatitis A Virus*.

*ANSI/ATCC ASN‐0002‐2022 Authentication of Human Cell Lines: Standardization of Short Tandem Repeat (STR) Profiling*.

*Pharmacopoeia of the People's Republic of China*.

*National Guide to Clinical Laboratory Procedures*.

## 3 Terms and Definitions

The following terms and definitions apply to this document:

### 3.1 Human Cortical Neural Progenitor Cells

A type of neural progenitor cells with limited proliferation and potential to specifically differentiate into cortical neurons and astrocytes.

### 3.2 Tumourigenicity

The property of a cell to form tumours when inoculated into an immunosuppressed animal model.

## 4 Abbreviations

The following abbreviations are applicable for this document.

B19: Human Parvovirus B19.

EBV: Epstein–Barr Virus.

HAV: Hepatitis A Virus.

HBV: Hepatitis B Virus.

HCMV: Human cytomegalovirus.

hCNPCs: Human Cortical Neural Progenitor Cells.

HCV: Hepatitis C Virus.

HIV: Human Immunodeficiency Virus.

HTLV: Human T‐lymphotropic Virus.

STR: Short Tandem Repeat.

## 5 General Requirements

### 5.1 Raw Materials

5.1.1 The collection of raw materials shall comply with the domestic legal and ethical requirements.

5.1.2 Based on the purpose of use, cell research and manufacture organizations shall establish and implement standards for donor evaluation.

### 5.2 Process and Information Management

5.2.1. Records of key factors affecting product quality in the processes of raw material acquisition, manufacturing, testing, shipping, and storage of cells shall be kept, and a unique identifier shall be established to ensure traceability of the processes.

5.2.2. The minimum retention period for records shall be clearly defined. The integrity of the records and the security of record storage shall be ensured.

## 6 Quality Requirements

### 6.1 Cell Morphology

hCNPCs under monolayer adherent culture exhibit uniform, translucent spindle shapes. While under 3D culture, they can spontaneously form dense, translucent spheres.

### 6.2 Cell Markers

6.2.1 Flow cytometry method: FOXG1 positive rate shall be ≥ 70%, PAX6 positive rate shall be ≥ 70%, NESTIN positive rate shall be ≥ 80%, PAX6 and NESTIN double positive rate shall be ≥ 60%.

6.2.2. Immunocytofluorescence method: FOXG1, PAX6, NESTIN shall be positive.

### 6.3 Cell Viability

The cell viability shall be ≥ 80% before cryopreservation, and ≥ 70% post‐thawing.

### 6.4 Cell Potency Indicators

6.4.1. After culture, hCNPCs shall be able to differentiate into cortical neurons and astrocytes. These cells shall have: a sum of human cortical neuron markers TBR1 and BRN2 positive rates ≥ 60%, and also express neuronal marker TUJ1 or MAP2, the positive rate of astrocyte marker AQP4 ≥ 5%, and also express astrocyte marker GFAP or S100β.

6.4.2. The electrophysiological activity can be detected by cell electrophysiology testing.

### 6.5 Chromosome Karyotype

The karyotype shall be normal as 46, XX or 46, XY.

### 6.6 Microorganisms

Fungi, bacteria, mycoplasma, HAV, HBV, HCV, HTLV, HIV, EBV, HCMV, B19 shall be negative.

### 6.7 Endotoxin

Endotoxin shall be < 5.0 EU/mL.

### 6.8 STR Authentication

The STR of hCNPCs shall be consistent with that of the original donor cells, and without contamination of heterologous cells.

### 6.9 Tumourigenicity

The tumourigenicity test result of hCNPCs obtained from either human tissue separation, or stem cell differentiation, or transdifferentiation, shall be negative.

## 7 Inspection Methods

### 7.1 Cell Morphology

Observe the morphology of cells grown under 2D or 3D conditions under an inverted microscope.

### 7.2 Cell Markers

7.2.1. Flow cytometry method: The method in Annex A in the Supporting Information can be followed.

7.2.2. Immunocytofluorescence method: The method in Annex B in the Supporting Information can be followed.

### 7.3 Cell Viability

The method in Annex C in the Supporting Information can be followed.

### 7.4 Cell Potency Assay

7.4.1. Neuronal differentiation potential assay: Refer to the method described in Annex D in the Supporting Information for the test.

7.4.2. Astrocyte differentiation potential assay: Refer to the method described in Annex E in the Supporting Information for the test.

7.4.3. Differentiated cell electrophysiology testing: Refer to the method described in Annex F in the Supporting Information for the testing.

Note: In addition to the methods described in Annex F in the Supporting Information, other calcium indicators with similar functions may also be considered for substitution. Furthermore, other validated electrophysiological detection methods, such as Multielectrode Array Assay (MEA) and patch‐clamp techniques, can also be adopted.

### 7.5 Chromosome Karyotyping

The method in the *Pharmacopoeia of the People's Republic of China (2020 Edition, Volume III)* shall be followed.

### 7.6 Microorganisms

7.6.1. Bacteria and Fungi: The ‘1101 Sterility Inspection Method’ in *Pharmacopoeia of the People's Republic of China (2020 Edition, Volume III)* shall be followed.

7.6.2. Mycoplasma: The ‘3301 Mycoplasma Inspection Method’ in *Pharmacopoeia of the People's Republic of China (2020 Edition, Volume III)* shall be followed.

7.6.3. HAV: The nucleic acid test method in WS 298 shall be followed.

7.6.4. HBV: The nucleic acid method in *National Guide to Clinical Laboratory Procedures* shall be followed.

7.6.5. HCV: The nucleic acid method in WS 213/T shall be followed.

7.6.6. HTLV: The nucleic acid method in *National Guide to Clinical Laboratory Procedures* shall be followed.

7.6.7. HIV: The nucleic acid method in WS 293/T shall be followed.

7.6.8. EBV: The nucleic acid method in *National Guide to Clinical Laboratory Procedures* shall be followed.

7.6.9. HCMV: The nucleic acid method in *National Guide to Clinical Laboratory Procedures* shall be followed.

7.6.10. B19: Refer to the method described in Annex G in the Supporting Information.

### 7.7 Endotoxins

The ‘1143 Bacterial Endotoxins Inspection Method’ in *Pharmacopoeia of the People's Republic of China (2020 Edition, Volume III)* shall be followed.

### 7.8 STR Authentication

*ANSI/ATCC ASN‐0002‐2022* shall be followed.

### 7.9 Tumourigenicity

For the preparation and quality control of animal cell substrates for biological product manufacturing and testing, the *“Tumourigenicity Test Method”* in *Pharmacopoeia of the People's Republic of China (2020 Edition, Volume III)* shall be followed.

## 8 Inspection Rules

### 8.1 Sampling Method and Quantities

8.1.1. Cells manufactured from the same process cycle, same cell source, same production line, same passage, and same methods are considered to be the same batch.

8.1.2. Three smallest units of packaging shall be randomly sampled from the same batch.

### 8.2 Release Testing

8.2.1. Each batch of products shall be subject to the quality inspection before release, and inspection reports shall be attached.

8.2.2. The quality inspection items shall include all the attributes specified in Section 6.

### 8.3 Verification Inspection

Verification inspection shall be performed by professional cell inspection institutions/laboratories as necessary.

### 8.4 Decision Rules

8.4.1. Products that pass all releasing tests in Section 6 are considered qualified. Products that fail to pass one or more release tests in this document are considered to be unqualified.

8.4.2. Products that pass all releasing tests in Section 6 by verification inspection are considered to be qualified. Products that fail to pass one or more release tests in this document are considered to be unqualified.

## 9 Instructions for Usage

The instructions for usage shall include, but not limited to:

- Product name,
- Passage number,
- Cell numbers,
- Production date
- Lot number,
- Manufacturer,
- Storage conditions,
- Shipping conditions,
- User manual,
- Execution standard number,
- Manufacturing address,
- Contact information,
- Postal code,
- Matters that need attention.

Note: Provide endotoxin testing result per user's request.

## 10 Labels

The label shall include but not limited to:

- Product name,
- Passage number,
- Cell number,
- Lot number,
- Manufacturer,
- Production date,
- Expiration date.

## 11 Shipping and STROAGE

### 11.1 Shipping

11.1.1. Based on the requirements of cell usage, the appropriate shipping method and condition shall be selected to ensure the biological characteristics, safety, stability, and efficacy of the cells.

11.1.2. Factors including but not limited to cell characteristics, containers used to contain the cells, shipping routes, shipping conditions, shipping equipment, shipping method, shipping risks and safeguard measures, shall be considered, in terms of the shipping of cells.

11.1.3. The control of transportation conditions should include, but not be limited to, temperature ranges, vibration, contamination‐free environment, equipment performance, proper packaging and so on.

11.1.4. Relevant inspection documents and technical guidance documents shall be provided according to the user's request.

11.1.5. Inspect packages in transit when necessary, and add new cold sources (such as dry ice or liquid nitrogen) when possible to maintain the appropriate shipping temperature.

### 11.2 Storage

11.2.1. Optimized cryopreservation procedures and methods shall be adopted to minimize damage to cells during the freeze–thaw process, ensuring that their normal functions will not be excessively affected by cryopreservation.

11.2.2. The cryopreservation information of the cells shall be recorded, including but not limited to:

- Name,
- Lot number,
- Quantity,
- Passage number,
- Cryopreservation date,
- Cryopreservation media components,
- Operator's name

11.2.3. The Storage Conditions of the Cells Shall Be Recorded, Including but Not Limited to:

- Storage condition,
- Storage date,
- Storage expiration date,
- Storage personnel.

## Author Contributions

An‐Xin Wang, Jing Fan, Jie Hao, Guang‐Zhu Zhang, Ai‐Jin Ma, Fang Ren, Yan‐Shung Xiao, Shi‐Jun Hu, Mei Tian, Hong Zhang contributed to conception and design. Tao Na, Ke‐Hua Zhang, Jia‐Ni Cao, Ting‐Ting Wu, Qiang Wei, Wei Qi Zhao, Lei Wang, Shuai‐Shauai Niu, Zhi‐Quan Yang and Tong‐Biao Zhao drafted and revised the manuscript. Ting Zhang, Gunag‐Jin Pan, Jun Yao, Ying Zhang, Yang Zhao, Yi Zhou, Li‐Jun Zhu, Qi‐Yuan Li, Wei‐Qi Zhang and Ka Li critically read and revised the manuscript.

## Funding

This work was supported by the National Key Research and Development Program of China, 2021YFA1101701; National Natural Science Foundation of China, Grant/Award Number: 82030049; Key Research and Development Program of Zhejiang Province, 2021C03090.

## Supporting information


**Data S1:** Supporting Information

## Data Availability

The data that support the findings of this study are available from the corresponding author upon reasonable request.

