# Supplementary material for: Human Cortical Neural Progenitor Cells
Source: Cell Prolif. 2026 Aug 4:e70266. Online ahead of print. doi: 10.1111/cpr.70266 (PMC13437111; doi:10.1111/cpr.70266)
Supplement: Supplementary file 1 [file CPR-9999-e70266-s001.docx]

**Annex A**

**（Informative）**

**DETECTION OF CELL MARKERS (FLOW CYTOMETRY)**

**A.1 Instruments and equipment**

A.1.1 Horizontal centrifuge

A.1.2 Electronic balance

A.1.3 Flow cytometer

A.1.4 Medical refrigerator

**A.2 Reagents**

A.2.1 Live-Dead cell staining dye

A.2.2 Fixation and permeabilization buffer

A.2.3 Human Fc receptor blocking solution

A.2.4 Forkhead box protein G1（FOXG1）and control antibody

A.2.5 Alexa Fluor 488 Mouse anti-Human PAX-6, control antibodies and secondary antibodies

A.2.6 PE Mouse anti-NESTIN and control antibody

A.2.7 SOX2 PE and control antibody

A.2.8 Bovine serum albumin（BSA）

A.2.9 Sodium chloride injection

A.2.10 Normal donkey serum（NDS）

A.2.11 Phosphate buffered saline（PBS）

**A.3 Test protocol**

A.3.1 Sample preparation

Collect single cell suspension by centrifuging at 300 g for 5 minutes, and then resuspend the cells gently in PBS（A.2.11）or sodium chloride injection（A.2.9）for 2 times by centrifuging at 500 g for 3 minutes, and discard the supernatant.

A.3.2 Live-Dead dye staining

Resuspend the cells in a working solution made by mixing the Live-Dead dye (A.2.1) and PBS (A.2.11) at a volume ratio of 1:3, then incubate in a medical refrigerator (A.1.4) for 30 minutes. After incubation, centrifuge at 300 g for 5 minutes using a horizontal centrifuge (A.1.1) and discard the supernatant. Then, resuspend the cells in a working solution made with BSA (A.2.8) and PBS (A.2.11), centrifuge at 300 g for 5 minutes using a horizontal centrifuge (A.1.1), and discard the supernatant.

A.3.3 Fixation and permeabilization

According to the instructions, fix and permeabilize the samples from step A.3.2, then aliquot the cells into seven independent samples.

A.3.4 Blocking

According to the instructions, block 5 samples of the samples with Human Fc Receptor Blocking Solution (A.2.3) and 2 samples with Normal Donkey Serum (A.2.10). These 2 samples will subsequently be stained with FOXG1 and control antibodies, as well as secondary antibodies.

A.3.5 Antibody incubation

According to the instructions, incubate the seven samples with the diluted antibodies （A.2.4、A.2.5、A.2.6、A.2.7）or corresponding isotype controls according to the manufacturer's instructions in a medical refrigerator (A.1.4). Then wash the cells once with the permeabilization buffer from the Fixation and Permeabilization Solution (A.2.2), then centrifuge at 500 g for 5 minutes using the horizontal centrifuge (A.1.1) and discard the supernatant.

A.3.6 Test

Resuspend the samples with PBS and proceed with detection on the flow cytometer (A.1.3) according to the manufacturer's instructions.

A.3.7 Gating principle

Gate the target population of cells and exclude cell debris on a scatter plot of forward scatter and side scatter. Then, based on the APC channel, encircle the viable cell population, excluding dead cells. Finally, establish the positive threshold based on the fluorescence intensity of the negative control group, and encircle the positive cell population within the viable cell population.

**A.4 Analysis of results**

Analyze the results using software according to manufacturer's instructions.

**Annex B**

**（Informative）**

**DETECTION OF CELL MARKERS (IMMUNOCYTOFLUORESCENT METHOD)**

**B.1 Instruments and equipment**

B.1.1 Inverted Fluorescence Microscope, excitation wavelength 488 nm, 594 nm

**B.2 Reagents**

B.2.1 Phosphate-buffered saline（PBS）: pH 7.4

B.2.2 Primary antibody：Rabbit anti-human PAX6, Mouse anti-human NESTIN, Rabbit anti-human FOXG1

B.2.3 Secondary antibody：Alexa 488 Donkey anti-Rabbit，Alexa 546 Donkey anti-Mouse

B.2.4 4% Paraformaldehyde（PFA）

B.2.5 Ultrapure water (sterile, endotoxin-free): cell culture grade

B.2.6 Polyethylene glycol octylphenyl ether (Triton X-100）

B.2.7 Anti-fluorescence quenching agent (with DAPI)

B.2.8 Normal donkey serum（NDS）

**B.3 Testing protocol**

B.3.1 Sample preparation and fixation

Put the cell suspension onto a matrix gel-coated cell slips (a round cell slip suitable for a 48-well plate can be used) and culture them for 4 days. Fix the cells using 4% paraformaldehyde (B.2.4) at room temperature for 30 minutes. Wash the cell samples with PBS (B.2.1) three times, each for 3 to 5 minutes. Meanwhile, use iPSCs with the same genetic background as the negative control.

B.3.2 Permeabilization and blocking

Permeate and block cells with PBS containing 0.25% Triton X100（B.2.6） and 3% NDS（B.2.8） at room temperature for 30 minutes.

B.3.3 Antibody incubation

Prepare the primary antibody working solutions for anti-PAX6 (B.2.2) and anti-NESTIN (B.2.2), anti-FOXG1 (B.2.2) and anti-NESTIN (B.2.2) using an antibody dilution solution (phosphate-buffered saline (B.2.1) containing 0.1% Triton X-100 (B.2.6) and 1% NDS (B.2.8)) according to the manufacturer's instructions. Incubate the antibodies with the prepared working solutions. Wash the cells with PBS (B.2.1) three times after incubation.

Prepare the second antibody working solutions according to the manufacturer's instructions. Incubate the antibodies with the prepared working solutions. Wash the cells with PBS (B.2.1) three times after incubation.

B.3.4 Nuclei staining, coverslips sealing

Transfer 5 μL of anti-fluorescence quenching agent (with DAPI) (B.2.7) to each cover slip for nuclei staining. Place the coverslip with cells onto the slide carefully without forming air bubbles. Seal the edges of coverslips with coverslip sealant.

B.3.5 Imaging

Adjust the fluorescence channel exposure time based on the signal intensity of the negative control samples. Choose at least three different visual fields randomly for imaging by using an inverted fluorescence microscope (B.1.1) with a 20× objective.

**B.4 Analysis of results**

Positive criteria: The cell nuclei exhibit round or oval blue fluorescence signals, NESTIN with fluorescence signals surrounding the blue round or oval signals is localized in the cytoplasm, FOXG1 and PAX6 with round or oval signals overlapping with the blue signals are localized in the nuclei, which is considered positive.

Negative criteria: The cell nuclei exhibit round or oval blue fluorescence signals, there are no other fluorescence signals, which is considered negative.

Invalid signal criteria: 1. There are no round or oval blue signals in the cell nucleus; 2. The NESTIN fluorescence signals do not surround the blue round or oval signals, or the FOXG1 and PAX6 signals do not overlap with the blue round or oval signals. If any of the above situations occur, the test is determined to be invalid, and the experiment must be repeated.

**Annex C**

**（Informative）**

**CELL VIABILITY TEST (CELL COUNTING METHOD)**

C.1 **Instruments and equipment**
    C.1.1 Automated cell counter
C.2 **Reagents**
    C.2.1 Dulbecco's phosphate-buffered saline（DPBS）
    C.2.2 0.2% Trypan blue solution
C.3 **Procedure**
    C.3.1 Preparing cell suspension

Harvest and suspend the cells with appropriate volume of DPBS (C.2.1).

C.3.2 Trypan blue staining

Evenly mix the Trypan blue solution (C.2.2) with the cell suspension at a volume ratio of 1:1.

C.3.3 Cell counting

Perform the test according to the manufacturer's instructions of the Automated Cell Counter (C.1.1), with each sample being measured in triplicate.

C.4 **Analysis of results**

The RSD of three independent measurements is ≤15%.

**Annex D**

**（Informative）**

**NEURONAL DIFFERENTIATION POTENTIAL ASSAY**

**D.1 Instruments and equipment**

D.1.1 CO_2_ incubator
D.1.2 Cryostat Microtome
D.1.3 Inverted Fluorescence Microscope

**D.2 Reagents**

D.2.1 Differentiation reagents
D.2.1.1 Neurobasal medium：Neurobasal Medium with 1× B-27（Vitamin A-free）and 1 mM GlutaMAX.
D.2.1.2 Y-27632 2HCl （ROCKi )
D.2.2 Testing reagents
D.2.2.1 Phosphate buffered saline (PBS): pH7.4.
D.2.2.2 Primary antibody：Mouse anti-Human MAP2, Mouse anti-Human TUJ1, Rabbit anti-Human TBR1, Goat anti-Human BRN2.
D.2.2.3 Secondary antibody：Alexa 546 Donkey anti-Mouse, Alexa 488 Donkey anti-Rabbit, Alexa 488 Donkey anti-Goat.
D.2.2.4 4% Paraformaldehyde (PFA)
D.2.2.5 Ultrapure water (sterile, endotoxin-free)：cell culture grade
D.2.2.6 Polyethylene glycol octylphenyl ether (Triton X-100）
D.2.2.7 Anti-fluorescence quenching agent (with DAPI)
D.2.2.8 Normal donkey serum（NDS）
D.2.2.9 Sucrose：Purity≥98%
D.2.2.10 O.C.T. Compound

**D.3 Testing protocol**

D.3.1 Cell Thawing
D.3.1.1 Prepare the neurobasal medium （D.2.1.1）with 10 µM ROCKi（D.2.1.2）.
D.3.1.2 Resuspend with pre-warmed neurobasal medium (D.2.1.1) containing ROCKi, centrifuge at 300 g for 3 minutes, and discard the supernatant.
D.3.1.3 Tap with index finger to disperse the cell clumps gently, then resuspend and disperse with neurobasal medium (D.2.1.1) containing 10 µM ROCKi (D.2.1.2).
D.3.1.4 After cell counting, proceed with the seeding for 3D brain Organoids.
D.3.2 Seeding and culturing of 3D brain Organoids.
D.3.2.1 Adjust the cell concentration to 2.0×10^5^ cells/mL with neurobasal medium (D.2.1.1) containing 10 µM ROCKi (D.2.1.2), and plate 4.0×10^4^ cells/well into an ultra-low attachment round-bottom 96-well cell culture plate.
D.3.2.2 Incubate at incubator（D.1.1）with 37℃, 5% CO_2_.
D.3.2.3 From day1-28, medium will be changed 1-2 times per week based on the consumption of the culture medium. The medium will be changed every other day from day 28, with each culture period lasting between 30 to 120 days.
D.3.3 Testing protocol
D.3.3.1 Fixation, dehydration, and sectioning

After 3D differentiation culture, fix the Organoids with 4% paraformaldehyde (D.2.2.4) at room temperature for 30 minutes, and wash with phosphate-buffered saline (D.2.2.1) three times, each for 3 to 5 minutes. Take the Organoids to a sucrose solution (D.2.2.9) until the samples sink to the bottom of the solution. Embed the Organoids using a cryosection embedding medium (D.2.2.10) and then proceed with cryosectioning using a cryostat microtome (D.1.2). Meanwhile, iPSCs with the same genetic background will be used as the negative control.

D.3.3.2 Blocking and permeabilization

Permeate and block cells with phosphate-buffered saline (D.2.2.1) containing 0.25% Triton X-100 (D.2.2.6) and 5% NDS (D.2.2.8) at room temperature for 60 minutes.

D.3.3.3 Antibody incubation

Prepare the primary antibody working solutions for anti-MAP2（D.2.2.2）/anti-TUJ1（D.2.2.2） and anti-TBR1（D.2.2.2）/anti-BRN2（D.2.2.2） using an antibody dilution solution (phosphate-buffered saline (D.2.2.1) containing 0.1% Triton X-100 (D.2.2.6) and 1% NDS (D.2.2.8)) according to the manufacturer's instructions. Incubate the antibodies with the prepared working solutions. Wash the cells with PBS (B.2.1) three times after incubation.

Prepare the secondary antibody working solutions（D.2.2.3）according to the manufacturer's instructions. Incubate the antibodies with the prepared working solutions. Wash the cells with PBS (D.2.2.1) three times after incubation.

D.3.3.4 Nuclei staining, coverslips sealing.

Transfer 20 µL of anti-fluorescence quenching agent (with DAPI) (D.2.2.7) to the slide with organoids cryosections for nuclei staining. Place the coverslip with cells onto the slide carefully without forming air bubbles. Seal the edges of coverslips with coverslip sealant.

D.3.3.5 Imaging

Adjust the fluorescence channel exposure time based on the signal intensity of the negative control samples, and image the entire section of organoids by the inverted fluorescence microscope (D.1.3).

**D.4 Analysis of results**

Positive criteria：The nucleus of the cell exhibits round or oval blue fluorescence signals, TUJ1 and MAP2 are subcellularly localized to the cell bodies and neurites, exhibiting linear fluorescence signals surrounding the blue round or oval signals, BRN2 and TBR1 are subcellularly localized to the nucleus, showing round or oval signals overlapping with the blue signals. which is considered negative.

Negative criteria: The nucleus of the cell exhibit round or oval blue fluorescence signals, there are no other fluorescence signals, which is considered negative.

Invalid signal criteria: 1. There are no round or oval blue signals in the cell nucleus. 2. The TUJ1 and MAP2 fluorescence signals do not surround the blue round or oval signals, or the BRN2 and TBR1 signals do not overlap with the blue round or oval signals. If any of the above situations occur, the test is determined to be invalid, and the experiment must be repeated.

**Annex E**

**（Informative）**

**ASTROCYTE DIFFERENTIATION POTENTIAL ASSAY**

**E.1 Instruments and equipment**

E.1.1 CO_2_ incubator
E.1.2 Cryostat Microtome

E.1.3 Inverted Fluorescence Microscope

**E.2 Reagents**

E.2.1 Differentiation reagents

E.2.1.1 Neurobasal medium：Neurobasal Medium with 1× B-27（Vitamin A-free）and 1 mM GlutaMAX.

Y-27632 2HCl （ROCKi )

E.2.2 Testing reagents

E.2.2.1 Phosphate buffered saline (PBS): pH 7.4

E.2.2.2 Primary antibody：Rabbit anti-Human GFAP, Rabbit anti-Human S100β, Rabbit anti-Human AQP4

E.2.2.3 Secondary antibody：Alexa 488 Donkey anti-Rabbit

E.2.2.4 4% Paraformaldehyde (PFA)

E.2.2.5 Ultrapure water (sterile, endotoxin-free)：cell culture grade

E.2.2.6 Polyethylene glycol octylphenyl ether (Triton X-100）

E.2.2.7 Anti-fluorescence quenching agent (with DAPI)

E.2.2.8 Normal donkey serum（NDS）

E.2.2.9 Sucrose：Purity≥98%

E.2.2.10 O.C.T. compound

**E.3 Testing protocol**

E.3.1 Cell thawing
E.3.1.1 Prepare the neurobasal medium （E.2.1.1）with 10 µM ROCKi（E.2.1.2）.
E.3.1.2 Resuspend with pre-warmed neurobasal medium (D.2.1.1) containing ROCKi, centrifuge at 300 g for 3 minutes, and discard the supernatant.
E.3.1.3 Resuspend with pre-warmed neurobasal medium (E.2.1.1) containing ROCKi （E.2.1.2）, centrifuge at 300 g for 3 minutes, and discard the supernatant.
E.3.1.4 After cell counting, proceed with the seeding for 3D brain Organoids.

E.3.1.5 Seeding and culturing of 3D brain organoids.

E3.1.6 Adjust the cell concentration to 2.0×10^5^ cells/mL with neurobasal medium (D.2.1.1) containing 10 µM ROCKi (D.2.1.2), and plate 4.0×10^4^ cells/well into an ultra-low attachment round-bottom 96-well cell culture plate.

E3.1.7 Incubate at incubator（D.1.1）with 37℃, 5% CO_2_.

E3.1.8 From day 1-28, medium will be changed 1-2 times per week based on the consumption of the culture medium. The medium will be changed every other day from day 28, with each culture period lasting between 30 to 120 days.

E.3.3 Testing protocol

E.3.3.1 Fixation, dehydration, and sectioning.

After 3D differentiation culture, fix the organoids with 4% paraformaldehyde (E.2.2.4) at room temperature for 30 minutes, and wash with phosphate-buffered saline (E.2.2.1) three times, each for 3 to 5 minutes. Take the organoids to a sucrose solution (E.2.2.9) until the samples sink to the bottom of the solution. Embed the organoids using a cryosection embedding medium (E.2.2.10) and then proceed with cryosectioning using a cryostat microtome (E.1.2). Meanwhile, iPSCs with the same genetic background will be used as the negative control.

E.3.3.2 Blocking and permeabilization

Permeate and block cells with phosphate-buffered saline (D.2.2.1) contain 0.25% Triton X-100 (E.2.2.6) and 5% normal donkey serum (E.2.2.8) at room temperature for 60 minutes.

E.3.3.3 Antibody incubation

Prepare the primary antibody working solutions for anti-AQP4 (E.2.2.2), anti-GFAP（E.2.2.2), anti-S100β (E.2.2.2）using an antibody dilution solution (phosphate-buffered saline (E.2.2.1) containing 0.1% Triton X-100 (E.2.2.6) and 1% NDS (E.2.2.8) according to the manufacturer's instructions. Incubate the antibodies with the prepared working solutions. Wash the cells with PBS (E.2.2.1) for three times after incubation.

Prepare the secondary antibody working solutions（E.2.2.3）according to the manufacturer's instructions. Incubate the antibodies with the prepared working solutions. Wash the cells with PBS (E.2.2.1) three times after incubation.

E.3.3.4 Nuclei staining, coverslips sealing.

Transfer 20 μL of anti-fluorescence quenching agent (with DAPI) (E.2.2.7) to the slide with organoids cryosections for nuclei staining. Place the coverslip with cells onto the slide carefully without forming air bubbles. Seal the edges of coverslips with coverslip sealant.

E.3.3.5 Imaging

Adjust the fluorescence channel exposure time based on the signal intensity of the negative control samples, and image the entire section of organoids by the inverted fluorescence microscope (E.1.3).

**E.4 Analysis of results**

Positive criteria: The cell nucleus exhibits round or oval blue fluorescence signals, AQP4, GFAP and S100β are subcellularly localized to the cell bodies and neurites, exhibiting linear fluorescence signals surrounding the blue round or oval signals, which is considered negative.

Negative criteria: The cell nucleus exhibits round or oval blue fluorescence signals, there are no other fluorescence signals, which is considered negative.

Invalid signal criteria: 1. There are no round or oval blue signals in the cell nucleus. 2. The AQP4, GFAP and S100β fluorescence signals do not surround the blue round or oval signals. If any of the above situations occur, the test is determined to be invalid, and the experiment must be repeated.

**Annex F**

**（Informative）**

**DIFFERENTIATED CELL ELECTROPHYSIOLOGY TESTING**

**(CALCIUM IMAGING METHOD)**

**F.1 Instruments and equipment**

F.1.1 CO_2_ incubator
F.1.2 Inverted Fluorescence Microscope

**F.2 Reagents**

F.2.1 Neurobasal medium：Neurobasal Medium with 1×B-27 (Vitamin A-free) and 1 mM GlutaMAX

F.2.2 Fluo-4, AM

F.2.3 Dimethyl sulfoxide（DMSO）

F.2.4 Hank's Balanced Salt Solution（HBSS）

**F.3 Testing protocol**

F.3.1 Sample preparation

Seed the cell suspension onto slides or glass-bottomed cell culture dishes, and culture with neurobasal medium (F.2.1) for 2D adherent differentiation for more than 14 days, or seed the cell suspension onto low attachment cell culture plates for 3D brain organoid suspension differentiation culture for more than 14 days.

F.3.2 Prepare of Fluo-4, AM storage solution

Dissolve Fluo-4, AM (F.2.2) with DMSO (F.2.3) to prepare a 1 mM storage solution.

F.3.3 Prepare of Fluo-4, AM working solution

Dilute the 1 mM Fluo-4, AM (F.2.2) storage solution to a working solution of 1-5 µM using HBSS (F.2.4).

F.3.4 Take out the differentiated samples ready for testing, remove the culture medium, and wash the cells three times with HBSS solution (F.2.4), then add the Fluo-4, AM working solution that covers the cells or brain organoids.

F.3.5 Incubate in a 37°C CO_2_ incubator (F.1.1) for 15-60 minutes, then remove the Fluo-4, AM working solution and wash the cells or brain organoids three times with HEPES-buffered solution (F.2.4).

F.3.6 Transfer of HBSS solution (F.2.4) and continue to incubate the cells or brain organoids in a 37°C CO_2_ incubator (F.1.1) for 30 to 40 minutes.

F.3.7 Record the cellular fluorescence signals using a fluorescence microscope (F.1.2) or other equipment, capturing a video of the calcium signals in cells or 3D brain organoids for 5 to 10 minutes at 494 nm excitation and 516 nm emission.

**F.4 Analysis of results**

Use intensity analysis software of image or video fluorescence (such as Image J) to analyze the changes in fluorescence intensity of cells in the video statistically and plot the curve graph. If at least one significant fluorescence signal peak can be observed within 1 minute, it is determined that the differentiated cells or brain organoids possess physiological functions.

**Annex G**

**（Informative）**

**B19 TESTING METHOD**

**G.1 Instrument and equipment**

G.1.1 qPCR machine

**G.2 Reagents**

G.2.1 Viral Extraction Kit

G.2.2 Premix

G.2.3 Primers

G.2.4 Probes

G.2.5 Positive plasmid control

**G.3 Procedure**

G.3.1 Prepare samples

Cell supernatant: No pretreatment is needed, take the sample for nucleic acid extraction directly.

Cell suspension: Take 1 mL of the cell suspension, take the supernatant for nucleic acid extraction after perform cell lysis and centrifugation.

G.3.2 Nucleic acid extracting

Nucleic acid extraction of the sample is performed using the Viral Nucleic Acid Extraction Kit (G.2.1).

G.3.3 Sample Testing

Transfer the probe premix (G.2.2), primers (G.2.3), probes (G.2.4), and template to the 8-well strip tubes for qPCR detection (G.1.1) and include a positive plasmid control (G.2.5) simultaneously.

G.4 Analysis of results

G.4.1 Validation of test effectiveness

If the Cp value is < detection limit, and there is a typical amplification curve, it is considered positive. Otherwise, it is negative.
